# Nutritional status and gait speed in a nationwide population-based sample of older adults

**DOI:** 10.1038/s41598-018-22584-3

**Published:** 2018-03-09

**Authors:** Joana Mendes, Nuno Borges, Alejandro Santos, Patrícia Padrão, Pedro Moreira, Cláudia Afonso, Rita Negrão, Teresa F. Amaral

**Affiliations:** 1 Department of Biomedicine, Biochemistry Unit, Faculty of Medicine, University of Porto, Rua Dr. Plácido da Costa, 4200-450 Porto, Portugal; 2 I3S - Institute for Research and Innovation in Health, University of Porto, Rua Alfredo Allen, 208, 4200-135 Porto, Portugal; 3 Faculty of Nutrition and Food Sciences, University of Porto, Rua Dr. Roberto Frias, 4200-465 Porto, Portugal; 4 CINTESIS - Centre for Health Technology and Services Research, Rua Dr. Plácido da Costa, 4200-450 Porto, Portugal; 5 EPIUnit, Institute of Public Health, University of Porto, Rua das Taipas, n° 135, 4050-600 Porto, Portugal; 6 The Research Centre in Physical Activity, Health and Leisure, University of Porto, Rua Dr. Plácido Costa, 91, 4200-450 Porto, Portugal; 7 UISPA-IDMEC, Faculty of Engineering, University of Porto, Rua Dr. Roberto Frias, 4200-465 Porto, Portugal

## Abstract

The association between nutritional status and gait speed remains unclear. This study described gait speed in older adults and quantified the association between overweight, obesity, undernutrition risk and gait speed. Gait speed as potential indicator of nutritional outcomes was also explored. A cross-sectional analysis was conducted in a population-based sample of 1,500 older adults ≥65 years old. Compared to “normal body mass index” women, odds ratio for a slow gait speed was approximately 2-fold higher in“overweight”, 4-fold higher in “obese” and 6-fold higher in women at “undernutrition risk”. “Undernutrition risk” category resulted from joining “undernutrition risk/undernutrition”. For men, these associations were in the same direction, but the odds ratio estimates halved. In women, identified gait speed cut-offs were 0.87 m/s for “obesity” and 0.79 m/s for “undernutrition risk”. In men, 0.94 m/s is the cut-off in which most older adults were correctly classified relative to “undernutrition risk”. About half of Portuguese older adults presented a gait speed ≤0.8 m/s. Overweight, obesity and undernutrition risk were directly and increasingly associated with slow gait speed, but approximately twice as high in women compared to men. Gait speed revealed potential utility in marking nutritional problems, but further investigation is recommended.

## Introduction

Older adults have a higher risk of age-related decline in functional performance and gait speed is used for functional assessment as it is a reliable, easy, quick, non-expensive and informative measurement^1,2^.

A poor nutritional status is also prevalent in the elderly^3,4^. In the oldest European population, overweight and obesity constitute a growing public health challenge^3^. In turn, undernutrition risk and undernutrition were observed in 60% of institutionalized older adults and in 11% of those living in the community^4^. Particularly in this age group, undernutrition can coexist with overweight and obesity^5^.

Functional performance is closely associated with nutritional well-being in older populations^3,6^. Handgrip strength is used to assess functional status for identification of undernutrition, but it is not appropriate for use in some populations with upper limb deformities and it cannot replace evaluation of lower extremity strength^7^. Gait speed could be a very informative measure in situations where it is not appropriate to measure handgrip strength. Misu *et al*. recently studied the association between undernutrition and gait speed among 204 community-dwelling older adults, and their results suggested that undernutrition status affects lateral trunk control during walking^8^. However, the association between undernutrition and gait speed has been scarcely investigated. Moreover, the association between overweight and gait speed remains to be explored and gait speed cut-offs associated with poor nutritional status are not yet defined.

This study aimed to describe gait speed in a nationwide sample of older adults and to quantify the association between overweight, obesity, undernutrition risk and gait speed. Furthermore, the usefulness of gait speed as a potential indicator of poor nutritional outcomes in older adults was also explored.

## Methods

### Study and sample design

A cross-sectional observational study was conducted in Portugal, in a cluster sample of 1,500 older adults ≥65 years old, between December 2015 and June 2016, in accordance with “The Nutrition UP 65 Study Protocol”^9^.

Data from the 2011 Census was used to obtain a nationally representative sample of Portuguese older adults in terms of sex, age, educational level and residence area defined in the Nomenclature of Territorial Units for Statistical purposes^9^. A random, stratified and cluster sampling method was applied. Three or more town councils with >250 inhabitants were randomly selected by each regional area. The potential participants were contacted directly at home by door knocking, through telephone or institutions, such as town councils and parish centres. Individuals were considered eligible if they had Portuguese nationality with current tax residence in Portugal, and if they were aged ≥65 years^9^. The initial sample was composed of 5% of older adults in nursing homes, a proportion similar to that of the Portuguese population^9^. The participant was considered a community-dwelling individual if he slept in his own house or in the house of a family member or a friend more than half of the days in the preceding month^9^. Written informed consent was obtained from older adults or their legally authorized representatives, depending on cognitive status.

### Data collection and variable definition

Eight interviewers worked on data collection during this period. All interviewers were previously trained, in order to improve intra and inter interviewer agreement, and the corresponding errors were obtained for anthropometric values. Intra-interviewer error ranged from 0.05% to 0.34% and inter-interviewer error varied between 0.19% and 1.48%. These values are considered acceptable for trained anthropometrists^9^.

Demographic data included information on sex, date of birth and education level determined by the number of completed school years.

Cognitive performance was assessed by the version of Mini Mental State Examination validated for the Portuguese population^9,10^. The cut-off scores for cognitive impairment were as follows: individuals with no education, <15 points; 1 to 11 years of years of school completed, <22 points; and >11 years of school completed, <27 points^9,10^. For individuals identified as presenting cognitive impairment, reported data was provided by a person close to the participant, such as a family member or caregiver.

Self-reported sitting time, data on chronic diseases and about self-perceived health status, as well as anthropometric and functional parameters, were measured and collected according to the standard procedures previously published in “The Nutrition UP 65 Study Protocol”^9^.

Poor nutritional status, namely overweight and obesity were identified using the BMI cut-offs of the World Health Organization criteria^11^. Undernutrition was identified using the Portuguese version of the Mini-Nutritional Assessment® - Short Form (MNA-SF)^9,12^. It is a valid nutritional screening tool for free-living and clinically relevant older populations, recommended by the European Society for Clinical Nutrition and Metabolism (ESPEN)^13^. The MNA-SF contains geriatric-specific assessment questions related to nutritional and health status, namely questions about possible food intake decline, weight loss during the previous three months, mobility, possible psychological stress or acute disease in the previous three months and neuropsychological problems^12^. Participants were considered undernourished if the final MNA-SF score was ≤7 points and they were considered at risk of undernutrition if the final MNA-SF score was between 8 and 11 points. Participants with a score ≥12 points were classified without undernutrition risk/undernutrition^12,13^. Considering the small proportion of participants classified as undernourished in the initial sample (n = 19, 1.3%), both undernourished and participants at risk of undernutrition were grouped together in a single category of “undernutrition risk/undernutrition”. From now on, for simplicity, “undernutrition risk/undernutrition” will be cited as “undernutrition risk”.

### Categories of nutritional status

In the initial sample, there were 37 (2.5%) cases of missing gait speed records and gait speed was not evaluated in 27 (1.8%) individuals due to mobility restrictions. In 11 (0.7%) cases, there were missing variables related to self-reported chronic diseases. The following categories of nutritional status were considered for 1,425 participants, corresponding to 95% of the total original dataset:

- “normal BMI”, which included participants with a BMI between 18.5 and 24.9 kg/m^2^ and without undernutrition risk according to a MNA-SF ≥ 12 points. This category was used as reference;
- “overweight”, which included participants with a BMI between 25.0 and 29.9 kg/m^2^ and without undernutrition risk according to a MNA-SF ≥ 12 points;
- “obesity”, which included participants with a BMI ≥ 30.0 kg/m^2^ and without undernutrition risk according to a MNA-SF ≥ 12 points;
- “undernutrition risk”, a category for participants with a MNA-SF score < 12 points. This category included participants with normal BMI, overweight and obesity.

### Gait speed

Gait speed was calculated for each participant using distance in meters and time in seconds^9,14^. It was obtained by dividing the distance travelled 4.6 m, on a flat and unobstructed path, by the time to cover that distance. The distance was marked on the floor with a visible tape. The study incorporated an additional distance of 2 m for acceleration and a further 2 m for deceleration^9,14^. The following instruction was given to the participant: “I will say ready, set, go. When I say go, walk at your normal and comfortable pace until I say stop”^9,14^. The walking time was registered with a stopwatch with a resolution of 0.01 s (School Electronic Stopwatch, Dive049, Topgim, Portugal).

### Ethics approval and consent to participate

This study was conducted according to the guidelines of the Declaration of Helsinki. Potential participants were contacted by the interviewer, who provided information about the study purposes and methodology, and invited them to participate. In cases of acceptance, cognitive performance was assessed by the version of Mini Mental State Examination validated for the Portuguese population with cut-offs depending on the educational level^10^. Individuals without cognitive impairment were asked to read and sign a duplicated informed consent form. If the participant was deemed to be cognitively impaired, two representatives were asked to read and sign the duplicated informed consent form. And so, in this way, written informed consent was obtained from participants or their legally authorized representatives.

The study protocol was approved by the Ethics Committee of Social Sciences and Health from Faculty of Medicine of University of Porto, Portugal (PCEDCSS – FMUP 15/2015) and by the Portuguese National Commission of Data Protection (9427/2015).

### Statistical analysis

A sensitivity analysis was carried out comparing the mean values of all variables between excluded and included individuals. The purpose of this analysis was to determine if the excluded older adults differed from those included in the present study, in order to evaluate potential results bias. Excluded individuals were older (mean age of 76.7 ± 7.9 years), compared to participants in the remaining sample (mean age of 74.9 ± 7.0 years), *p* = 0.032. However, in relation to all other variables, including gait speed, BMI and nutritional status, significant differences were not found.

Characteristics of the sample were presented stratified by sex and by gait speed. The median of the sample distribution (0.8 m/s) was used to establish a cut-off of gait speed, because there are no validated gait speed cut-offs for nutritional outcomes. Categorical variables were presented as counts and proportions and were compared using the chi-square test. The normality of variables distribution was examined with the Kolmogorov-Smirnov test. Means and standard deviation values were calculated and compared using the Student’s *t*-test and Anova test.

A logistic regression model was used to estimate the probability of a gait speed ≤0.8 m/s according to poor nutritional status, stratified by sex. Chi-square test for trend was estimated. Odds ratio and 95% confidence intervals were estimated adjusting for age, height, mid-arm muscle circumference, Mini Mental State Examination score, self-reported sitting time and for the number of chronic diseases, as continuous variables.

Receiver operating characteristic (ROC) curves were constructed to evaluate the performance of gait speed adjusted for height (gait speed/height) to identify poor nutritional outcomes, particularly obesity and undernutrition. The areas under the curves (AUCs) and their 95% confidence intervals were also calculated. The AUC should be >0.5 in an effective screening test, an AUC = 0.5 indicates that the screening test is no better than chance. Sensitivities, specificities and positive likelihood ratios (LR), for describing the ability of gait speed to identify poor nutritional outcomes, were also calculated. Likelihood ratio is an alternative method for evaluating the value of a diagnostic test. It expresses “the probability that a given test result would occur in a person with the target disorder divided by the probability that the same result would occur in a person without that disorder” and positive LR is attained through the formula: LR = sensitivity/(1 − specificity)^15^. Values between 0 and 1 show the decrease in post-test probability of having poor nutritional outcomes according to low gait speed values^15^. Values higher than 1 show the increase of post-test probability of having poor nutritional outcomes according to low gait-speed values^15^.

Results were considered significant when *p* < 0.05.

## Results

Participants’ age ranged from 65 to 100 years and gait speed ranged from 0.18 to 2.25 m/s, with a median of 0.84 m/s.

In this nationwide sample, 16.6% of participants were classified with normal BMI, 44.6% with overweight and 38.9% with obesity. In the total sample, 15.2% of individuals were included in the “undernutrition risk” category and among these participants, 27.2% had a normal BMI, 35.5% were overweight and 37.3% were obese.

Characteristics of the sample are summarized in Table 1. According to the gait speed cut-off (0.8 m/s), statistically significant differences in BMI were observed among women, but not in men. Women with slow gait speed had higher BMI than women with high gait speed values (*p* < 0.001).

**Table 1:** Characteristics of the sample according to sex and to gait speed.

	Women (n = 834)			Men (n = 591)		
	Gait speed ≤ 0.8 m/s	Gait speed > 0.8 m/s	*p*	Gait speed ≤ 0.8 m/s	Gait speed > 0.8 m/s	*p*
n (%)	404 (48.4)	430 (51.6)		186 (31.5)	405 (68.5)	
**Socio-demographic parameters**						
Age (years), mean (SD)	78.5 (7.1)	72.4 (5.9)	<0.001	77.4 (7.4)	72.7 (5.8)	<0.001
Education (years), n (%)						
no formal schooling	96 (23.8)	48 (11.2)	<0.001	30 (16.1)	21 (5.2)	<0.001
1–4 years of completed school	273 (67.6)	300 (69.8)		128 (68.8)	280 (69.1)	
>4 years of completed school	35 (8.7)	82 (19.1)		28 (15.1)	104 (25.7)	
**Cognition and subjective health**						
Mini-Mental State Examination, n (%)						
without cognitive impairment	356 (88.1)	417 (97.0)	0.001	169 (90.9)	396 (97.8)	<0.001
with cognitive impairment	48 (11.9)	13 (3.0)		17 (9.1)	9 (2.2)	
Self-reported sitting time (hours/day), mean (SD)	6.7 (3.1)	4.4 (2.4)	<0.001	6.3 (3.2)	4.5 (2.3)	<0.001
Number of chronic diseases, mean (SD)	4.4 (2.1)	4.1 (2.1)	0.046	3.3 (2.0)	2.9 (1.8)	0.026
**Self-perceived health status** ^**†**^ **, n (%)**						
good/very good	76 (18.9)	150 (35.1)	<0.001	54 (29.0)	177 (43.7)	<0.001
moderate	199 (49.4)	223 (52.2)		89 (47.8)	194 (47.9)	
bad/very bad	128 (31.8)	54 (12.6)		43 (23.1)	34 (8.4)	
**Anthropometry and functionality**						
Weight (Kg), mean (SD)	69.3 (13.5)	68.4 (11.6)	0.263	76.6 (12.7)	77.7 (11.4)	0.201
Height (cm), mean (SD)	150.0 (6.1)	152.6 (5.9)	<0.001	163.1 (6.8)	165.7 (6.6)	<0.001
Body mass index (Kg/m^2^), mean (SD)	30.7 (5.4)	29.3 (4.5)	<0.001	28.8 (4.4)	28.3 (3.7)	0.134
Mid-arm muscle circumference (cm), mean (SD)	22.9 (3.0)	22.4 (3.7)	0.033	23.6 (3.0)	25.1 (5.2)	0.001
Waist circumference (cm), mean (SD)	99.6 (12.5)	94.9 (11.2)	<0.001	104.7 (11.7)	101.8 (9.7)	0.002
Calf circumference (cm), mean (SD)	35.4 (3.9)	35.7 (3.4)	0.195	35.6 (3.3)	36.2 (3.5)	0.022
Handgrip strength (Kgf), mean (SD)	15.8 (4.6)	20.5 (4.9)	<0.001	25.9 (7.5)	33.1 (8.7)	<0.001
**Categories of nutritional status, n (%)**						
normal body mass index^‡^	21 (5.2)	59 (13.7)	<0.001	24 (12.9)	63 (15.6)	<0.001
overweight^§^	117 (29.0)	169 (39.3)		68 (36.6)	199 (49.1)	
obesity^¶^	172 (42.6)	148 (34.4)		58 (31.2)	110 (27.2)	
undernutrition risk^#^	94 (23.3)	54 (12.6)		36 (19.4)	33 (8.1)	

^**†**^Information was not reported by four individuals (0.3%).

^**‡**^BMI between 18.5 and 24.9 kg/m^2^ (WHO criteria) and without undernutrition risk/undernutrition (MNA-SF ≥ 12 points).

^**§**^BMI between 25.0 and 29.9 kg/m^2^ (WHO criteria) and without undernutrition risk/undernutrition (MNA-SF ≥ 12 points).

^¶^BMI ≥ 30.0 kg/m^2^ (WHO criteria) and without undernutrition risk/undernutrition (MNA-SF ≥ 12 points).

^#^MNA-SF score was <12 points.

*Abbreviations*: BMI, body mass index; MNA-SF, Mini-Nutritional Assessment-Short Form; SD, standard deviation; WHO, World Health Organization.

Compared to women with “normal BMI”, the odds ratio for a gait speed ≤0.8 m/s was approximately 2-fold higher in women with “overweight”, 4-fold higher in women with “obesity” and 6-fold higher in women with “undernutrition risk” (*p* for trend: <0.001) (Table 2). For male older adults, these associations were in the same direction but the odds ratio estimates halved (*p* for trend: 0.001) (Table 2). These estimates were independent of factors such as age, height, mid-arm muscle circumference, Mini Mental State Examination score, self-reported sitting time and number of chronic diseases (Table 2).

**Table 2:** Crude and adjusted odds ratio of slow gait speed (≤0.8 m/s), stratified by sex, according to categories of nutritional status.

	Gait speed ≤ 0.8 m/s					
	Women			Men		
	Crude OR (95% CI)	Adjusted^#^ OR (95% CI)	*p* ^*^	Crude OR (95% CI)	Adjusted^#^ OR (95% CI)	*p* ^*^
Normal body mass index^†^	1	1	<0.001	1	1	0.001
Overweight^‡^	1.95 (1.12–3.38)	2.42 (1.13–5.18)		1.88 (0.52–1.55)	1.08 (0.47–1.67)	
Obesity^§^	3.27 (1.89–5.63)	3.97 (1.63–9.67)		1.38 (0.79–2.44)	1.84 (0.81–4.19)	
Undernutrition risk^¶^	4.89 (2.68–8.91)	5.98 (2.46–14.53)		2.86 (1.47–5.58)	2.96 (1.31–6.64)	

^**†**^BMI between 18.5 and 24.9 kg/m^2^ (WHO criteria) and without undernutrition risk/undernutrition (MNA-SF ≥ 12 points).

^**‡**^BMI between 25.0 and 29.9 kg/m^2^ (WHO criteria) and without undernutrition risk/undernutrition (MNA-SF ≥ 12 points).

^**§**^BMI ≥ 30.0 kg/m^2^ (WHO criteria) and without undernutrition risk/undernutrition (MNA-SF ≥ 12 points).

^¶^MNA-SF score was <12 points.

^#^Adjusted for age, height, mid-arm muscle circumference, Mini-Mental State Examination, self-reported sitting time and for the number of chronic diseases (all as continuous variables).

^*^p-value of chi-square for trend.

*Abbreviations:* BMI, body mass index; CI, confidence intervals; MNA-SF, Mini-Nutritional Assessment-Short Form; OR, odds ratio; WHO, World Health Organization.

Participants with “normal BMI” had a gait speed mean of 0.95 m/s (women) and 0.99 m/s (men), those with “overweight” had a mean of 0.89 m/s (women) and 1.01 m/s (men), participants with “obesity” had a mean of 0.79 m/s (women) and 0.94 m/s (men), participants at “undernutrition risk” had a mean of 0.72 m/s (women) and 0.84 m/s (men) (*p* < 0.001 for each sex, according to nutritional status categories). For both sexes, no statistically significant differences were found between the gait speed of participants without obesity and gait speed of obese participants in the “undernutrition risk” category.

**Figure 1:**
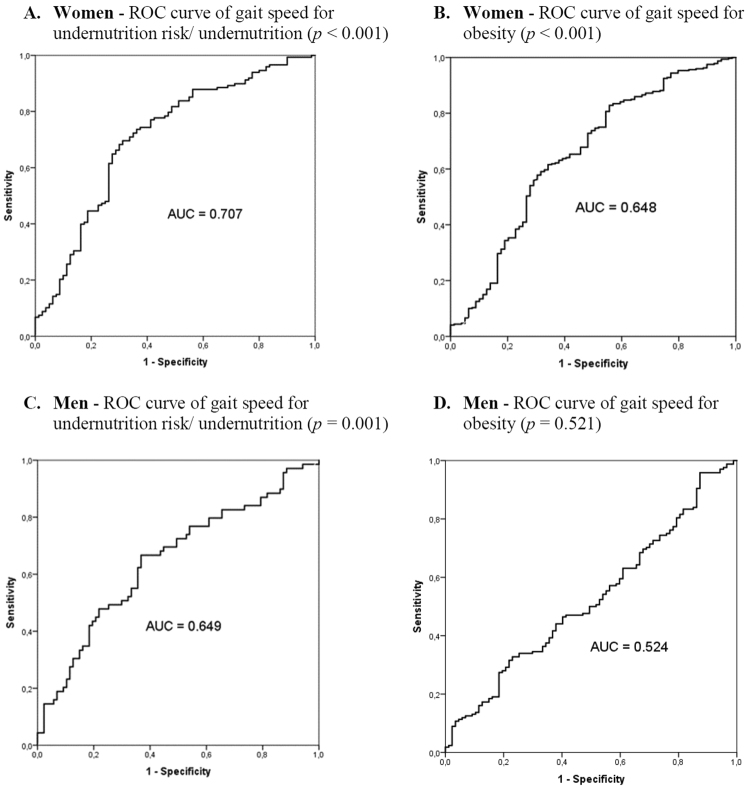
Sex-specific ROC curves of gait speed (adjusted for height) for undernutrition risk/undernutrition (**A** and **C**) and for obesity without undernutrition risk (**B** and **D**).

Results on the performance of gait speed adjusted for height to identify poor nutritional outcomes are displayed in Table 3 and Fig. 1. For women with “obesity” and at “undernutrition risk”, LR were >1 and AUCs of ROC curves were >0.5 and statistically significant (*p* < 0.001) (Table 3 and Fig. 1A,B). In women, identified gait speed cut-offs were 0.87 m/s for “obesity” and 0.79 m/s for “undernutrition risk” (Table 3).

**Table 3:** Gait speed cut-offs, adjusted for height, and diagnostic values of risk of poor nutritional status, for women and men.

	Sensitivity	Specificity	AUC*	95% CI	LR	Cut-off for gait speed (m/s)	Cut-off for time to walk 4.6 m (s)
**Women**							
Undernutrition risk^†^	0.696	0.675	0.707	(0.635–0.780)	2.14	0.79	5.82
Obesity^‡^	0.756	0.450	0.648	(0.576–0.719)	1.37	0.87	5.29
**Men**							
Undernutrition risk^†^	0.768	0.460	0.649	(0.561–0.736)	1.42	0.94	4.89

^**†**^Mini-Nutritional Assessmen-Short Form (MNA-SF) score was < 12 points.

^**‡**^Body mass index ≥ 30.0 kg/m^2^ (World Health Organization criteria) and without undernutrition risk/undernutrition (MNA-SF ≥ 12 points).

*Abbreviations*: AUC, area under the curve; CI, confidence interval; LR, likelihood ratio. *p < 0.05.

For men at “undernutrition risk”, the gait speed of 0.94 m/s was the value in which most older adults were correctly classified (Table 3). A sensitivity of 0.768, a specificity of 0.460, a LR > 1 and an AUC > 0.5 were estimated (*p* = 0.001) (Table 3 and Fig. 1C). The ROC curve was not statistically significant for men with “obesity” (*p* = 0.521) (Fig. 1D).

## Discussion

In the present study, the association between overweight, obesity, undernutrition risk and gait speed in older adults was explored and results have shown that they are directly and increasingly associated with slow gait speed.

According to the literature, mobility impairment is defined as a usual gait speed ≤0.8 m/s, because this cut-off predicts disability and reduced overall survival^16^. This value coincides approximately with the median of gait speed in our sample and was used to establish a cut-off. Consequently, it is worrying that such a large proportion of older adults revealed mobility impairment. Changes related to the aging process and poor nutritional outcomes can explain the low gait speed values found among our participants. Firstly, it is known that in older populations, the general decrease in muscle strength due to loss of motor neurons, muscle fibers and aerobic capacity, is associated with a decreased gait speed and consequent adaptation^17^. This study sample is particularly prone to these disabilities due to the participants’ old age, with a median of 74 years. Secondly, obesity has been consistently associated with an increased risk of deterioration in physical functionality among older adults^18^. Therefore, the high proportion of participants with overweight and obesity can also explain gait speed results. Even among participants who were at “undernutrition risk”, more than half were also overweight or obese.

Overweight and obesity exerts an excessive load on body and knee joints leading on shorter step length that prompts slower gait speed^19^. Mechanisms that underlie the association between obesity and gait speed may also be mediated by several cytokines secreted from adipose tissue and consequent chronic inflammation^20,21^. Recently, potential obesity-related differences in neural activity during ambulation in older adults were reported^22^. Otherwise, a slow gait speed can also lead to obesity by decreased physical activity and increased sedentary lifestyle^23^. It is important to note that present analysis relies on cross-sectional data and temporal relationships could not be inferred. Therefore, we explored both possible scenarios: the association between nutritional status and gait speed, and gait speed as a potential functional marker of these problems.

In turn, undernutrition is strongly related with low muscle mass and strength which can also explain a low gait performance^24,25^. Recent research demonstrated that undernutrition can affect lateral trunk control during walking^8^. Among community-dwelling older adults, gait speed was also associated with MNA score, used to assess nutritional status^26^. These findings corroborate our results concerning the association between undernutrition and low gait speed. However, it is important to emphasize that the “undernutrition risk” category included overweight/obese participants.

The coexistence of undernutrition with obesity is now a reality which has been connected to higher dependence on the activities of daily living and with aggravation of mobility impairment among community-dwelling older adults^5,27^. Undernutrition is often associated with sarcopenia which is characterized by an age-related decline in muscular strength and functional ability^27^. It has been suggested that the association between low muscle mass and functional decline is explained by underlying muscle strength^28^. The coexistence of sarcopenia with obesity is also now a reality named sarcopenic obesity, which may carry the cumulative risk derived from each of the two individual body composition phenotypes^29,30^. Despite these facts, a sensitivity analysis conducted in our sample did not present statistically significant differences between gait speed of participants at undernutrition risk without obesity and gait speed of those at undernutrition risk with obesity.

According to the Academy of Nutrition and Dietetics/American Society for Parenteral and Enteral Nutrition, there is no single parameter that is definitive for recognition of undernutrition in adults and older adults^6^. The identification of two or more of a set of six defined characteristics is recommended for undernutrition diagnosis, among which handgrip strength is included as functional measure^6^.

Handgrip strength has been showing a good predictive validity identifying those patients with a higher length of hospital stay^31,32^. However, handgrip strength cannot replace the assessment of functionality of lower limbs or the assessment of activities of daily living. This study is a first step towards knowing if gait speed may be an alternative functional measure, when the measurement of handgrip strength is not possible.

In the present sample, overweight, obesity and undernutrition were directly and increasingly associated with slower gait speed, but more strongly in women than in men. In line with our results, it was previously reported that elevations of several baseline adiposity measures were associated with a decline in gait speed, but also more strongly in women than in men^33^. Gender differences may be explained by the differences in body composition^34,35^. Body mass index use as a surrogate indicator of adiposity can be prone to errors because fat mass and fat free mass are not differentiated^34^, even though present results have been adjusted for the mid-arm muscle circumference. In men, the contribution of skeletal muscle mass to the total body weight is higher than in women, while in women, the proportion of fat mass/skeletal muscle mass is generally higher than in men^35,36^. Consequently, the association between overweight/obesity assessed through BMI and low gait speed can become more evident in women than in men.

In addition, although there is no data on the frequency of hip osteoarthritis in our sample, differences according to sex in the relation between obesity, hip damage and gait speed^37,38^ are also a potential explanation of why in the present study obesity was more strongly associated with low gait speed in women than in men.

Regarding the performance of gait speed to identify older adults presenting poor nutritional outcomes, although sensitivities higher than 65% were found, the specificities were relatively low. These sensitivity values are within the acceptable ranges for nutritional assessment tools^39^ and can be regarded as a strength because a sensitive test is more useful at an early stage of nutritional assessment, since it identifies high proportion of those older adults who present poor nutritional outcomes^39^. Because specific tests are useful in confirming a diagnosis that has been suggested by other procedures^39^ the low specificity cannot be interpreted as a major problem, however it is obviously desirable to have a test that is both highly sensitive and highly specific^39^.

Nevertheless, this is an exploratory study that displays preliminary results concerning the potential capacity of gait speed to identify poor nutritional outcomes. Further research should be conducted, comparing the ability of slow gait speed relative to a standard functional assessment methodology, in order to identify these poor nutritional outcomes.

This study is one of the first to explore gait speed as afunctional measure associated with poor nutritional outcomes in older adults, not only with undernutrition, but also with overweight and obesity. Imbalances in groups sizes may have been a limitation. However, in the present sample, other hypotheses of stratification according to nutritional outcomes were not feasible because of the high prevalence of overweight and obesity.

## Conclusion

About half of Portuguese older adults presented a gait speed ≤0.8 m/s. Overweight, obesity and undernutrition risk were directly and increasingly associated with a slow gait speed, but this association was approximately twice as high in women compared to men. Gait speed revealed potential utility as a marker of nutritional problems, but further investigation is required to understand its ability to be part of a screening or assessment method of nutritional status dysfunctions.
